# Impact of Hypothermic Oxygenated Machine Perfusion on Immune Cell Clearance in Liver Transplantation: Enhancing Graft Function and Post-Transplant Outcomes

**DOI:** 10.3390/jcm14010127

**Published:** 2024-12-29

**Authors:** Dominik Thomas Koch, Malte Schirren, Severin Jacobi, Hanno Nieß, Bernhard Willibald Renz, Jens Werner, Markus Otto Guba, Dionysios Koliogiannis

**Affiliations:** Department of General, Visceral and Transplantation Surgery, LMU University Hospital, LMU Munich, Marchioninistr. 15, 81377 Munich, Germany; dominik.koch@med.uni-muenchen.de (D.T.K.);

**Keywords:** liver transplantation, hypothermic oxygenated machine perfusion, immune cell clearance, immune cell characterization

## Abstract

**Background:** Hypothermic oxygenated machine perfusion (HOPE) has emerged as a critical innovation in liver transplantation (LTx), offering significant protection against ischemia-reperfusion injury (IRI). This study focuses on quantifying and characterizing immune cells flushed out during HOPE to explore its effects on graft function and post-transplant outcomes. **Materials and Methods:** Fifty liver grafts underwent end-ischemic HOPE. Perfusate samples were collected at three time points: at the start of perfusion, after 10 min, and at the end of perfusion. The samples were analyzed to quantify and characterize immune cells, assessing the effectiveness of HOPE in reducing cellular debris and its impact on graft quality. **Results:** The primary perfusate contained significant concentrations of immune cells, mainly segmented neutrophils, lymphocytes, and monocytes. After 10 min of perfusion, outflow cell concentration decreased by over 95%, and by the end of perfusion, a more than 99% reduction was observed. **Conclusions:** HOPE effectively reduces immune cell concentrations in liver grafts, suggesting a mechanism for improved graft function and reduced post-transplant complications. These findings support the continued use and optimization of HOPE in LTx.

## 1. Background

Liver transplantation (LTx) remains the only life-saving therapeutic strategy for patients with end-stage liver disease. However, the scarcity of eligible liver grafts has led to the increased use of marginal donors. The extended donor criteria for marginal liver grafts encompass an advanced donor age, prolonged cold storage, liver steatosis, obesity, prolonged intensive care unit stay, donation after circulatory death, and elevated liver-associated lab parameters [1]. These grafts require meticulous organ procurement and intensive perioperative care due to their heightened sensitivity to ischemia-reperfusion injury (IRI) [2]. This process occurs when oxygenated blood re-enters the liver after a period of ischemia. Intracellular energy depletion during hypoxic conditions is followed by oxidative and microcirculatory stress during reperfusion. This leads to cell death, the accumulation of damage-associated molecular patterns (DAMPs), and inflammation, triggering a complex inflammatory response involving both donor- and recipient-derived innate and adaptive immune cells [3,4,5]. These mechanisms lead to a cascade of complications, including post-reperfusion syndrome (PRS), early graft dysfunction, and increased morbidity. Marginal grafts are particularly prone to IRI as they exhibit impaired hepatic microcirculation and lower tolerance to oxidative stress [6].

To mitigate these risks, hypothermic oxygenated machine perfusion (HOPE) has gained widespread adoption since its clinical introduction in 2010 [7,8]. HOPE provides continuous perfusion and oxygenation of the liver graft at low temperatures, reducing metabolic demands, repaying the oxygen dept of hypoxia, and preventing the accumulation of harmful reactive oxygen species (ROS) that contribute to IRI [9,10,11]. These mechanisms reduce mitochondrial injury, nuclear damage, Kupffer cell activation, and endothelial stress. Consequently, compared to static cold storage (SCS), HOPE has been associated with improved graft function, fewer biliary complications, better electrolyte balance, better hemodynamic stability, and lower incidences of post-reperfusion syndrome (PRS) [12,13,14]. While HOPE’s benefits in preserving graft function are well documented, its impact on immune cell dynamics within the graft is less understood.

The negative effects of transfused immune cells have been known for decades, particularly in the context of leukocyte-contaminated blood products. Studies have shown that leukocyte transfusion can result in adverse outcomes, including febrile nonhemolytic transfusion reactions, alloimmunization, transfusion-associated immunosuppression, the transmission of infectious diseases, and thromboembolic diseases [15,16,17]. These findings underline the capacity of immune cells to provoke unintended inflammatory and immunological responses in the recipient.

In solid organ transplantation, the transfer of donor-derived immune cells embedded within the graft presents a parallel risk and can adversely impact the recipient’s organism. Donor livers harbor a substantial repertoire of resident immune cells [18]. These cells occupy the sinusoids and the space of Disse and can be mobilized to the intravascular space, including during SCS. This accumulation can be exacerbated by insufficient flushing during organ procurement. Insufficient flushing contributes to early allograft dysfunction (EAD) and poorer patient and graft survival [19]. These cells can intensify inflammatory responses upon reperfusion if not adequately removed, contributing to PRS, graft dysfunction, and even acute organ rejection [20].

Given the potential for donor-derived immune cells to negatively affect transplant outcomes, understanding the role of HOPE in immune cell clearance is crucial. This study aims to quantify and characterize the immune cells washed out during end-ischemic HOPE to assess and elucidate a potential mechanism for its beneficial effects on graft function and post-transplant outcomes.

## 2. Materials and Methods

### 2.1. Study Design

This prospective study was conducted in 2023 at the Department of General Visceral and Transplantation Surgery, LMU University Hospital, LMU Munich. Fifty liver grafts that underwent end-ischemic HOPE were included. The study aimed to evaluate the impact of HOPE on immune cell clearance and its subsequent effect on graft quality.

### 2.2. HOPE Protocol

Liver grafts were procured after brain death and preserved using the standard protocol of static cold storage (SCS) in Histidine−tryptophan−ketoglutarate (HTK) solution during transport. Upon arrival at the transplant center, the grafts were prepared for implantation and connected to the LiverAssist^®^ device (XVIVO, Groningen, The Netherlands, and Göteborg, Sweden). The perfusion process used a University of Wisconsin machine perfusion solution (UW-MPS) at temperatures between 8 and 12 °C. The portal vein was cannulated without pre-flushing, and perfusion was initiated at a pressure of 3 to 5 mmHg [14]. The portal vein pressure was adjusted in this pressure range to achieve a portal venous flow of 100 to 150 mL/minute.

After collecting the first samples, the hepatic artery was prepared and connected to the machine, if a cannulation of the hepatic artery was feasible. The hepatic artery was perfused with a pressure of 25 mmHg (dual-HOPE).

HOPE was run for a mean of 178.4 min (standard deviation 93.12 min). At the end of machine perfusion, the grafts were flushed with HTK to wash out UW-MPS immediately before implantation, as UW-MPS is not meant to be flushed in the recipient’s blood circulation.

### 2.3. Sample Collection

Perfusate samples were collected at the following three key time points during the HOPE process:

1. Primary perfusate: immediately after the initiation of perfusion directly out of the vena cava.
2. 10 min: after 10 min of continuous perfusion.
3. End of machine perfusion: just before the end of the machine perfusion process, before flushing the grafts with HTK to prepare the graft for implantation.

These samples were then analyzed for cell count, and a cell characterization was performed.

### 2.4. Cell Quantification and Characterization

Cell counts and characterization were performed using standard hematology techniques. Red blood cells, as well as nuclear cells, including polymorphonuclear cells, were counted. The focus was on quantifying and characterizing polymorphonuclear cells, which are known to contribute to inflammatory processes.

All analyses were performed at the Institute of Laboratory Medicine, LMU University Hospital, LMU Munich.

### 2.5. Statistical Analysis

Statistical analysis and chart design were performed using GraphPad Prism 10 (GraphPad Software, San Diego, CA, USA). A *p*-value of less than 0.05 was considered statistically significant.

## 3. Results

All cell measurements during HOPE were performed in 2023. For 50 liver grafts, the cell counts in the primary perfusate were measured. Measurements at the time point “10 min” were performed for 35 of the 50 liver grafts, and measurements at the time point “end of machine perfusion” were performed for 47 of the 50 liver grafts. For 44 of these 50 liver grafts, enough cells for a detailed cell characterization were available in the primary perfusate samples.

### 3.1. Cell Quantification

**Figure 1:**
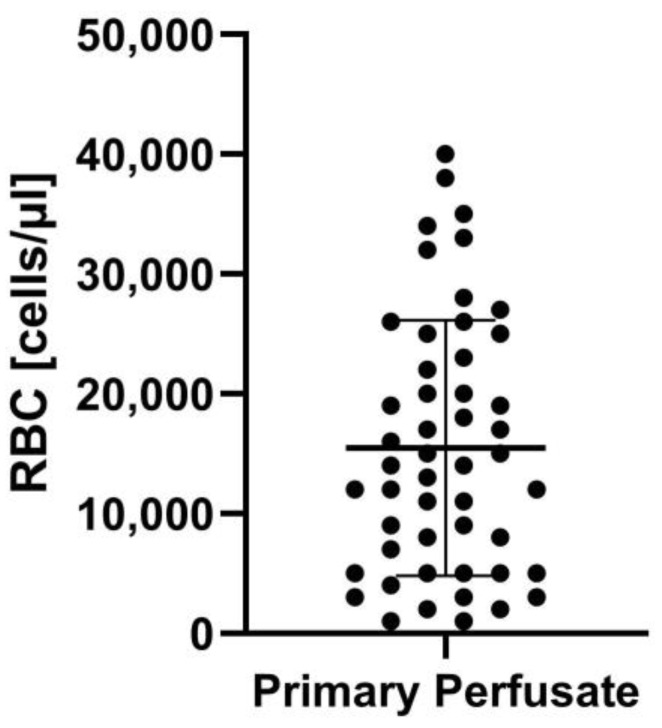
Red blood cell (RBC) quantification of primary perfusate.

Initial perfusate samples revealed a mean red blood cell (RBC) concentration of 15,480 RBC/µL, ranging from 1000 RBC/µL to 40,000 RBC/µL, and a standard deviation of 10,658 (Figure 1).

**Figure 2:**
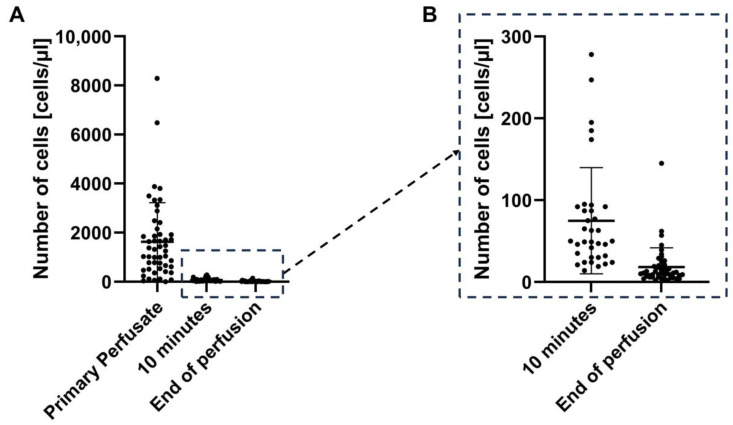
(**A**): Cell quantification of primary perfusate and at the time points “10 min” and “end of perfusion”; (**B**): smaller scale to focus on the cell quantification of the time points “10 min” and “end of perfusion”.

The nuclear cells measured in the initial perfusate samples had an outflow cell concentration of 1626 cells/µL, with a range from only 1 to 8286 cells/µL (Figure 2A). The standard deviation was 1595.

After 10 min of machine perfusion, this outflow concentration dropped to a mean of 74.94 cells/µL, ranging from 14 to 278 cells/µL, and a standard deviation of 64.89 (Figure 2A). By the end of perfusion, the mean outflow concentration further decreased to a mean of 18.06 cells/µL, ranging from 1 to 145 cells/µL, with a standard deviation of 23.34 (Figure 2A). This represents a more than 99% reduction in cell concentration from the initial to the final perfusate (*p* < 0.0001; paired *t*-test, two-tailed, 47 pairs). A focus on the outflow cell concentrations at the time points “10 min” and “end of perfusion” can be found in Figure 2B.

The outflow concentration of 23.34 cells/µL at the end of machine perfusion with a total volume of 3 L of machine perfusate results in a mean total cell count of 70.0 million cells flushed out of the liver grafts.

### 3.2. Cell Characterization

**Figure 3:**
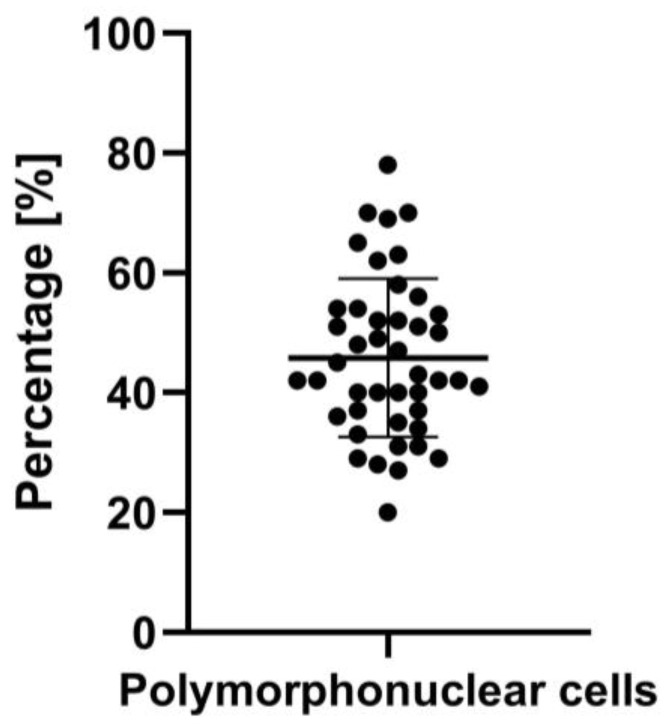
Percentage of polymorphonuclear cells in the primary perfusate.

In 44 out of 50 cases, a detailed cell characterization was performed. The mean percentage of polymorphonuclear cells in the primary perfusate was 45.82%, with a standard deviation of 13.22% (Figure 3). The minimum was 20%, and the maximum was 78%.

Among the polymorphonuclear cells, precursor cells of the granulopoiesis, namely promyelocytes, myelocytes, metamyelocytes, and banded neutrophils, were detected. Also, normoblasts, precursors of the erythropoiesis, were detected. Segmented neutrophils, basophils, eosinophils, lymphocytes, plasma cells, monocytes, and macrophages were detected, being representatives of the peripheral blood and peripheral tissue cells. Last, atypical lymphocytes (lymphoid cells) were detected.

**Figure 4:**
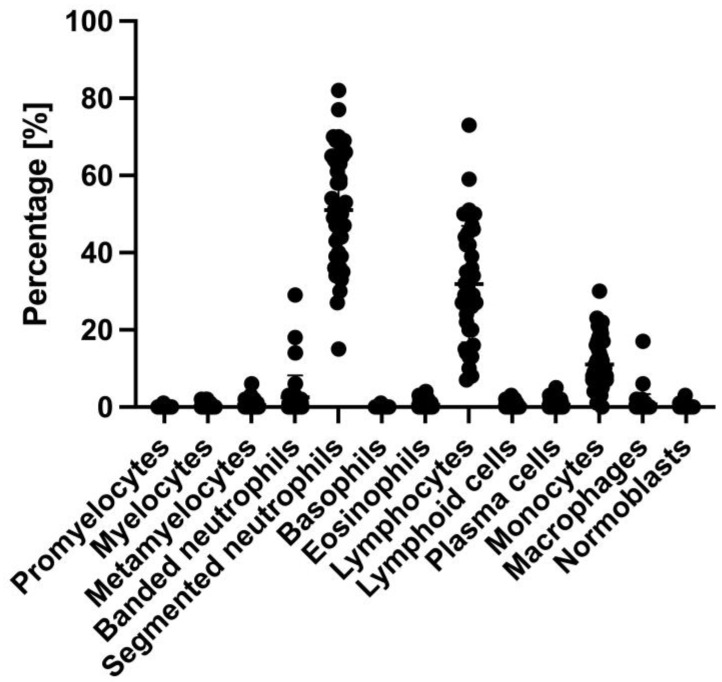
Cell characterization of the primary perfusate.

**Figure 5:**
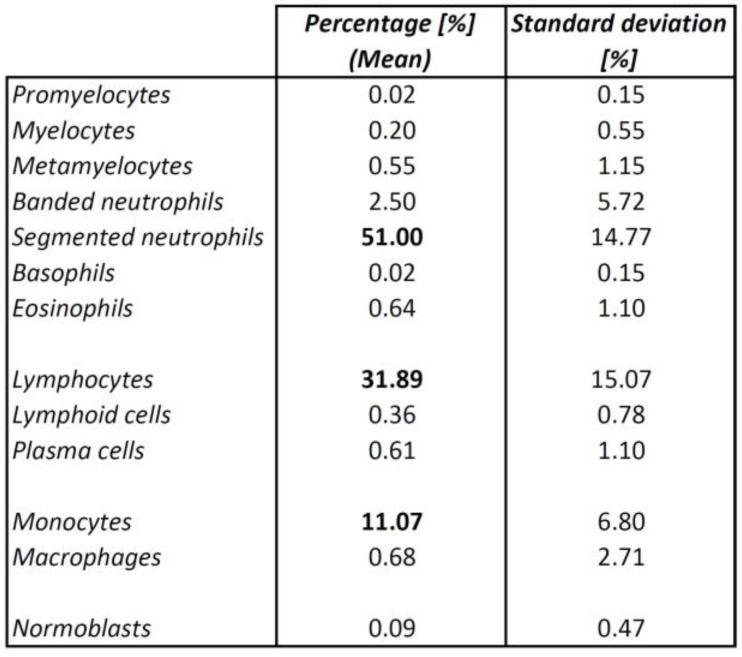
Cell characterization of the primary perfusate: Mean percentages of cells and standard deviation; the mean percentages of the predominant cell populations are printed in bold.

The primary immune cell types found in the primary perfusate were segmented neutrophils (mean percentage 51.00%), lymphocytes (31.89%), and monocytes (11.07%) (Figure 4; mean percentages and standard deviation in Figure 5).

Smaller populations of the described other cell types were only detected for some of the 44 cases. Promyelocytes were detected in one case with a percentage of 1%. Myelocytes were detected in 6 cases, metamyelocytes in 12 cases, banded neutrophils in 18 cases, basophils in 1 case with a rate of 1%, eosinophils in 15 cases, atypical lymphocytes (lymphoid cells) in 9 cases, plasma cells in 14 cases, macrophages in 7 cases, and normoblasts in 2 cases. The respective mean percentages are, therefore, small (Figure 4; mean percentages and standard deviation in Figure 5).

## 4. Discussion

Our study demonstrates the significant clearance of red blood cells and nuclear cells, including immune cells, during hypothermic oxygenated machine perfusion (HOPE). The samples gathered from the primary perfusate out of the vena cava immediately after starting HOPE allowed for detailed immune cell characterization. We identified segmented neutrophils, lymphocytes, and monocytes as the predominant populations in the primary perfusate.

By the end of perfusion, immune cell concentrations were reduced by over 99%, highlighting HOPE’s effectiveness in removing circulating cells and debris from the liver graft. The mean outflow concentration of 1626 cells/µL in the primary perfusate was diluted to a mean outflow concentration of 23.34 cells/µL at the end of machine perfusion.

This quantified clearance of cells illustrates that despite aortic perfusion during the liver procurement and a possible subsequent back table portal vein perfusion immediately after explanation, a substantial number of cells and debris remain within the liver vessels or are mobilized during the static cold storage (SCS). Especially, the variability in red blood cell (RBC) and nuclear cell concentrations, reflected by high standard deviations, suggests inconsistencies in donor organ perfusion during procurement. While detailed information about the in situ organ perfusion during organ procurement is not available, inadequate organ perfusion during procurement is associated with increased rates of early allograft dysfunction (EAD), as well as poorer graft and patient survival [19]. HOPE subsequently compensates prior suboptimal perfusion and reduces one out of many factors that negatively influence graft quality and patient outcomes, while other factors like donor age, cold ischemia time, or steatosis cannot be influenced upon arrival in the recipient center.

The significant reduction in immune cell concentrations observed in this study suggests that HOPE effectively eliminates cells that might otherwise contribute to complications such as post-reperfusion syndrome (PRS), EAD, and passenger lymphocyte syndrome. By minimizing immune cells and cell debris, HOPE likely reduces inflammatory responses triggered upon reperfusion, thereby improving graft function.

The clearance of these cells and debris is expected to lower the risk of EAD, often driven by inflammation and immune activation. Post-transplant inflammation, which can lead to tissue injury, involves both donor- and recipient-derived innate and adaptive immune cells [3]. Reducing this inflammation enhances allograft outcomes.

Previous studies indicate that HOPE-treated grafts exhibit better hemodynamic stability and fewer complications like PRS, resulting in more favorable immediate postoperative outcomes [12,13,14]. This improvement may partly be attributed to the reduction of post-transplant inflammation through the clearance of immune cells and cell debris.

The implications of these findings extend beyond the operating room. By reducing the immune cell burden in the liver graft, HOPE may contribute to a smoother postoperative course, with fewer complications related to inflammation, such as systemic inflammatory response syndrome (SIRS) or acute rejection episodes. Additionally, reducing immune cells could potentially reduce the need for aggressive immunosuppression, thereby minimizing the long-term side effects of these medications, such as infections, malignancies, and metabolic disorders. This could result in improved long-term graft survival and a better quality of life for transplant recipients.

While this study focused on liver transplantation, the principles of HOPE could be broadly applicable to other solid organ transplants, such as the kidneys, lungs, and heart. The ability of HOPE to reduce immune cell concentrations and cell debris and improve organ quality could lead to the widespread adoption of this technique across various types of transplantation. Furthermore, the insights gained from this study could inform future innovations in organ preservation technologies, particularly those aimed at minimizing ischemia-reperfusion injury (IRI) and immune activation.

Previous studies have established the benefits of machine perfusion in reducing ischemia-reperfusion injury and improving post-transplant outcomes [12,13,14]. Our study builds on this body of work by providing quantitative data on immune cell clearance, offering a mechanistic explanation for the improved outcomes observed in HOPE-treated grafts. These findings are consistent with and expand upon the existing literature, reinforcing the role of HOPE as a superior method of organ preservation compared to static cold storage.

Despite the promising findings, this study has several limitations. While sufficient to demonstrate substantial reductions in immune cell concentrations, the sample size may not capture the full variability seen in clinical practice. Additionally, the study focused primarily on short-term outcomes, and long-term follow-up data are needed to fully understand the impact of HOPE on graft survival and patient outcomes over time. Future studies should also explore the application of HOPE in a wider range of organs and patient populations to assess its generalizability. Last, the data do not allow any conclusion on the ongoing re-homing or mobilization of immune cells during machine perfusion—the latter could pave the way for ex-situ graft leukodepletion.

While the study quantified the reduction of immune cell concentrations, it did not deeply explore the specific molecular mechanisms involved in immune cell clearance by HOPE and how this process impacts IRI and post-transplant immune responses. For investigating the molecular mechanisms of immune cell clearance, it would be conceivable to design experiments using Lgr5+ liver cells that express the liver stem cell marker Lgr5 [21,22] or to use extracted liver stem cells of donor organs to culture organoids [23] in co-culture with immune cells.

Building on the findings of this study, future research should also aim to investigate the long-term effects of HOPE on graft survival, chronic rejection, and patient quality of life. Studies involving larger and more diverse patient populations could help validate the results and support the broader implementation of HOPE. Additionally, exploring the potential of HOPE in other organ transplants and refining perfusion protocols to further optimize immune cell clearance will be important next steps in advancing the field of organ preservation.

Finally, integrating HOPE with other emerging technologies, such as ex vivo organ conditioning and targeted molecular therapies, could open new avenues for improving transplant outcomes. Combining these approaches may offer even greater protection against IRI and immune-mediated damage, paving the way for safer and more effective transplantation procedures.

## 5. Conclusions

This observational study demonstrates that HOPE effectively reduces immune cell concentrations in liver grafts. These findings support the continued use and optimization of HOPE in LTx. However, further studies and comparisons with SCS are necessary to understand the contribution of HOPE-associated immune cell clearance to graft function and post-transplant complications.

## Data Availability

Data will be made available upon reasonable request and can be acquired by approaching the corresponding author.

## Abbreviations

DAMP	damage-associated molecular pattern
EAD	Early allograft dysfunction
HOPE	Hypothermic oxygenated machine perfusion
HTK	Histidine−tryptophan−ketoglutarate solution
IRI	Ischemia-reperfusion injury
LTx	Liver transplantation
PRS	Post-reperfusion syndrome
RBC	Red blood cell
ROS	Reactive oxygen species
SCS	Static cold storage
SIRS	systemic inflammatory response syndrome
UW-MPS	University of Wisconsin machine perfusion solution
